# Some Person Unknown

**Published:** 1856-12

**Authors:** 


*Some Person Unknown.*—A number of gentlemen have ordered copies of the "Physicians' Diary" from us, before it was ready for delivery, but the list was mislaid. We have been able to recover all the orders, *except one*, from our file of letters. This one gentleman will, of course, miss his "Diary." If he will take the trouble to inform us of his address, it will be promptly sent.

